# Mitochondrial genetic variants associated with bipolar disorder and Schizophrenia in a Japanese population

**DOI:** 10.1186/s40345-023-00307-6

**Published:** 2023-07-21

**Authors:** Ryobu Tachi, Kazutaka Ohi, Daisuke Nishizawa, Midori Soda, Daisuke Fujikane, Junko Hasegawa, Ayumi Kuramitsu, Kentaro Takai, Yukimasa Muto, Shunsuke Sugiyama, Kiyoyuki Kitaichi, Ryota Hashimoto, Kazutaka Ikeda, Toshiki Shioiri

**Affiliations:** 1 School of Medicine, Gifu University, Gifu, Japan; 2 Department of Psychiatry, Gifu University Graduate School of Medicine, Gifu, Japan; 3 Department of General Internal Medicine, Kanazawa Medical University, Ishikawa, Japan; 4 Addictive Substance Project, Tokyo Metropolitan Institute of Medical Science, Tokyo, Japan; 5 Laboratory of Pharmaceutics, Department of Biomedical Pharmaceutics, Gifu Pharmaceutical University, Gifu, Japan; 6 Department of Pathology of Mental Diseases, National Institute of Mental Health, National Center of Neurology and Psychiatry, Kodaira, Tokyo Japan

**Keywords:** Bipolar disorder, Schizophrenia, Mitochondria, Genetic variant, Family history

## Abstract

**Background:**

Bipolar disorder (BD) and schizophrenia (SZ) are complex psychotic disorders (PSY), with both environmental and genetic factors including possible maternal inheritance playing a role. Some studies have investigated whether genetic variants in the mitochondrial chromosome are associated with BD and SZ. However, the genetic variants identified as being associated are not identical among studies, and the participants were limited to individuals of European ancestry. Here, we investigate associations of genome-wide genetic variants in the mitochondrial chromosome with BD, SZ, and PSY in a Japanese population.

**Methods:**

After performing quality control for individuals and genetic variants, we investigated whether mitochondrial genetic variants [minor allele frequency (MAF) > 0.01, *n* = 45 variants) are associated with BD, SZ, and PSY in 420 Japanese individuals consisting of patients with BD (*n* = 51), patients with SZ (*n* = 172), and healthy controls (HCs, *n* = 197).

**Results:**

Of mitochondrial genetic variants, three (rs200478835, rs200044200 and rs28359178 on or near *NADH dehydrogenase*) and one (rs200478835) were significantly associated with BD and PSY, respectively, even after correcting for multiple comparisons (*P*_*GC*_=0.045–4.9 × 10^− 3^). In particular, individuals with the minor G-allele of rs200044200, a missense variant, were only observed among patients with BD (MAF = 0.059) but not HCs (MAF = 0) (odds ratio=∞). Three patients commonly had neuropsychiatric family histories.

**Conclusions:**

We suggest that mitochondrial genetic variants in NADH dehydrogenase-related genes may contribute to the pathogenesis of BD and PSY in the Japanese population through dysfunction of energy production.

**Supplementary Information:**

The online version contains supplementary material available at 10.1186/s40345-023-00307-6.

## Introduction

Bipolar disorder (BD) and schizophrenia (SZ) are severe and chronic psychotic disorders (PSY) with a lifetime prevalence of approximately 1% (Grande et al. 2016; Saha et al. 2005). BD and SZ have high heritability of approximately 80% (McGuffin et al. 2003; Sullivan et al. 2003). To date, the largest-scale genome-wide association studies (GWASs) have reported 64 and 287 genetic loci associated with BD and SZ, respectively (Mullins et al. 2021; Trubetskoy et al. 2022). Moreover, these PSY extensively share genetic factors with BD and SZ (Ohi et al. 2022b; Ruderfer et al. 2018; Smeland et al. 2020), though each disorder has disorder-specific genetic factors (Ruderfer et al. 2018). Most GWASs have focused on autosomal and/or sex chromosomes, and the genetic etiology for BD and SZ remains to be fully resolved.

Higher rates of PSY are observed in offspring of maternal probands compared to offspring of paternal probands with BD (McMahon et al. 1995) and SZ (Verge et al. 2011; Wolyniec et al. 1992). Therefore, several studies have investigated genetic associations with PSY comprising BD and SZ of variants in the chromosome of mitochondria, the energy-producing structures within cells, but not in autosomal and sex chromosomes (Gonçalves et al. 2018; Hagen et al. 2018; Kato et al. 2000; Mosquera-Miguel et al. 2012; Munakata et al. 2004; Ryu et al. 2018; Sequeira et al. 2012; Xu et al. 2017; Zhang et al. 2014). However, the mitochondrial genetic variants examined in these studies were selected based on a candidate gene approach, and the examined mitochondrial genetic variants were inconsistent among studies (Gonçalves et al. 2018; Hagen et al. 2018; Kato et al. 2000; Munakata et al. 2004; Ryu et al. 2018; Sequeira et al. 2012). Furthermore, results were also inconsistent among studies because of relatively small sample sizes.

To date, a limited number of studies have investigated genetic associations with BD and SZ of genome-wide genetic variants in the mitochondrial chromosome (*n* = 220–465 variants) (Gonçalves et al. 2018; Hagen et al. 2018; Hudson et al. 2014; Sequeira et al. 2012) (summarized in Supplementary Table 1). These studies have identified several mitochondrial genetic variants associated with BD (rs28357375 and rs28357968 in 965 patients with BD and 3,938 controls (Sequeira et al. 2012) and SZ (rs527236209, rs869096886 and rs1599988 in 4,778 patients with SZ and l5,819 controls (Gonçalves et al. 2018), rs2854131, rs2853503, rs2853504, rs193302985 and rs2853506 in 2,019 patients with SZ and 15,302 controls (Hudson et al. 2014), rs193302985 and rs2853506 in 2,538 patients with SZ and 23,743 controls (Hagen et al. 2018), rs3937033 and rs2857291 in 1,137 patients with SZ and 3,938 controls (Sequeira et al. 2012). Furthermore, a study investigated associations of mitochondrial genetic variants with PSY in BD and SZ and identified some genetic variants (rs2857291, rs28357968, rs28380140, rs3088053 and rs2853497) related to PSY in 2,102 patients with PSY and 3,938 controls (Sequeira et al. 2012). However, these genetic variants are not identical among studies. Furthermore, the investigated individuals were limited to those of European ancestry. The minor allele frequencies (MAF) of most of these genetic variants are < 1% in the Asian population (Supplementary Table 1).

Mitochondria are essential intracellular organelles that harbor original haploid genomes. Mitochondria play a crucial role in oxidative phosphorylation. In humans, thirteen proteins related to oxidative phosphorylation are synthesized from an approximately 16,600 bp of the mitochondrial genome. On the other hand, mitochondria are sources of free radicals. As their DNA does not have histones or effective repair mechanisms, it is particularly susceptible to certain stress-induced damage. Mitochondrial dysfunction can cause dysfunction of the central nervous system, which demands high energy. Associations between mitochondrial dysfunctions and PSY have been investigated (Nishimura et al. 2021; Wu et al. 2019), with hypotheses that mitochondrial dysfunctions affect synaptic, energic and metabolic pathways, resulting in SZ and BD (Giménez-Palomo et al. 2021; Morris et al. 2020; Steckert et al. 2010).

Although most previous studies have targeted European populations (Gonçalves et al. 2018; Hagen et al. 2018; Hudson et al. 2014; Sequeira et al. 2012), we hypothesized that common mitochondrial genetic variants are associated with BD and SZ in both European and Asian populations but that some mitochondrial genetic variants might be associated with BD and SZ only in Asian populations. In this study, we investigated possible associations of genome-wide genetic variants (MAF > 0.01, *n* = 45 variants) in the mitochondrial chromosome with BD, SZ, and PSY in a Japanese population. Furthermore, we investigated common characteristics among the patients who carried specific mitochondrial genetic variants.

## Methods

### Sample description

The subjects consisted of 51 patients with BD (23 males/28 females; mean age ± SD: 54.5 ± 16.8 years), 172 patients with SZ (77 males/95 females; 45.3 ± 14.0 years), and 197 HCs (129 males/68 females; 35.6 ± 13.8 years). The patients were recruited from both outpatient and inpatient populations at Kanazawa Medical University Hospital and related psychiatric hospitals. These participants were recruited from the Schizophrenia Non-Affected Relative Project (SNARP) (Kataoka et al. 2020; Ohi et al. 2017, 2019, 2020a, b, 2021) and the Bipolar & Schizophrenia Network on Intermediate Phenotypes in Japan (B-SNIP-J) (Ohi et al. 2022a; Ohi et al., 2022c) All SZ patients (*n* = 172) and HCs (*n* = 197) who participated in a previous study (Ohi et al. 2020b, 2022b) were included in the current study. Each patient was diagnosed by at least two trained psychiatrists based on unstructured clinical interviews, medical records, and clinical conferences. The patients were diagnosed according to the criteria in the fifth edition of *Diagnostic and Statistical Manual of Mental Disorders* (DSM-5). HCs were recruited through local advertisements and from among hospital staff at Kanazawa Medical University and were also evaluated using the nonpatient version of Structured Clinical Interview for DSM-IV (SCID) to exclude individuals who had current or past contact with psychiatric services, who had received psychiatric medication or who had a family history of any neuropsychiatric diseases among second-degree relatives. Imipramine equivalents of total antidepressants (IMI-eq), diazepam equivalents (DZ-eq), chlorpromazine equivalents of total antipsychotics (CPZ-eq), and biperiden equivalents of total antiparkinsonian drugs (BPD-eq) were calculated based on a previous study (Inada and Inagaki 2015). Current clinical symptoms were evaluated using the 17-item Hamilton Rating Scale for Depression (HAMD-17), the Young Mania Rating Scale (YMRS), and the Positive and Negative Syndrome Scale (PANSS). The premorbid IQ was evaluated using JART (Matsuoka et al. 2006), which is the Japanese version of the National Adult Resulting Test (NART). The demographic variables among the three groups are summarized in Table 1. Written informed consent was obtained from all participants after the procedures had been thoroughly explained. This study was performed in accordance with the Declaration of Helsinki from the World Medical Association and was approved by the Research Ethical Committees of Gifu University and Kanazawa Medical University.

**Table 1:** Demographic characteristics of patients with BD, patients with SZ, and HCs.

	HCs	BD	SZ		
Variables	(*n* = 197)	(*n* = 51)	(*n* = 172)	*P* values (*F* or *χ*^*2*^)	*post hoc*
Age (years)	35.6 ± 13.8	54.5 ± 16.8	45.3 ± 14.0	**6.79 × 10** ^**− 18**^ **(43.5)**	HCs < SZ < BD
Sex (male/female)	129/68	23/28	77/95	**1.25 × 10** ^**− 4**^ **(18.0)**	-
Education (years)	16.2 ± 2.4	13.5 ± 3.0	12.5 ± 2.2	**7.60 × 10** ^**− 41**^ **(116.2)**	HCs > BD > SZ
Estimated premorbid IQ	108.9 ± 7.4	100.8 ± 11.3	98.2 ± 11.0	**7.29 × 10** ^**− 22**^ **(55.3)**	HCs > BD,SZ
IMI-eq (mg/day)	-	45.9 ± 99.2	4.9 ± 27.0	**2.06 × 10** ^**− 6**^ **(23.8)**	-
DZ-eq (mg/day)	-	6.1 ± 10.4	6.2 ± 11.3	0.96 (< 0.1)	-
CPZ-eq (mg/day)	-	152.9 ± 213.3	523.3 ± 511.4	**9.80 × 10** ^**− 7**^ **(27.7)**	-
BPD-eq (mg/day)	-	0.1 ± 0.6	0.8 ± 2.3	**0.045 (4.1)**	-
Age at onset (years)	-	37.0 ± 16.2	27.0 ± 11.0	**9.19 × 10** ^**− 7**^ **(25.5)**	-
DOI (years)	-	17.5 ± 14.1	18.1 ± 12.7	0.76 (0.1)	-
HAMD-17	-	8.5 ± 5.9	-	-	-
YMRS	-	1.7 ± 4.5	-	-	-
PANSS positive symptoms	-	-	16.5 ± 6.2	-	-
PANSS negative symptoms	-	-	19.4 ± 7.0	-	-

HCs, healthy controls; BD, bipolar disorder; SZ, schizophrenia; IQ, intelligence quotient; IMI-eq, imipramine equivalents of total antidepressants; DZ-eq, diazepam equivalents; CPZ-eq, total antipsychotic dosage in chlorpromazine equivalents; BPD-eq, biperiden equivalents of total antiparkinsonian drugs; DOI, duration of illness; HAMD-17, 17-item Hamilton Rating Scale for Depression; YMRS, Young Mania Rating Scale; PANSS, Positive and Negative Syndrome Scale. Complete demographic information was not obtained for all subjects (number of participants for which the estimated premorbid IQ was available: HCs, *n* = 177; BD, *n* = 46; SZ, *n* = 168; number of participants for which the HAMD-17 was available: BD, *n* = 47). Means ± SDs are shown. *P* values < 0.05 are shown in boldface, and *post hoc* analysis was performed

### Genotyping and quality control

A detailed description of the genotyping and quality control (QC) applied in the study has been reported previously (Ohi et al. 2020a, b, 2021). Briefly, venous blood was collected from the participants, and genomic DNA was extracted from whole-blood samples. Genotyping was performed using Infinium OmniExpressExome-8 v1.4 or v1.6 BeadChips (Illumina, San Diego, CA, USA). After QC for removing subjects with high missing genotype rates (> 95%) and sex chromosome anomalies and genetic variants deviating from Hardy-Weinberg equilibrium (HWE) (*p* < 1.0 × 10^− 5^) or having a low MAF < 0.01 (Ohi et al. 2020b, 2022b), only genetic variants in the mitochondrial chromosome were extracted from the whole-genome genotyping data using PLINK v1.90 beta. As the sample sizes for each diagnostic group were different, the different sample size, especially smaller sample size, would affect the SNP QC including lower MAF (e.g., 0.001–0.05) and excess SNPs might be excluded for combined diagnostic comparison group (PSY vs. HCs). Thus, we also performed SNP QC procedure for each diagnostic comparison group (BD vs. HCs, and SZ vs. HCs). For mitochondrial genetic variants (*n* = 80), those that deviated from HWE (*p* < 1.0 × 10^− 5^), had a low MAF < 0.01, or had a poor genotype call rate (< 0.95) were excluded from each diagnostic comparison group (BD vs. HCs, and SZ vs. HCs) as well as a combined diagnostic comparison group (PSY vs. HCs).

### Statistical analyses

Statistical analyses for demographic variables were performed using IBM SPSS Statistics 28.0 software (IBM Japan, Tokyo, Japan). Differences in continuous variables, such as age and years of education, among diagnostic groups were analyzed using analysis of variance (ANOVA). Differences in categorical variables, such as sex, were analyzed using Pearson’s *χ*^*2*^ test. Genetic analyses were performed in PLINK. Differences in allele frequency between PSY and HCs, BD and HCs, and SZ and HCs were analyzed using the *χ*^*2*^ test. To test for the existence of genetic structure in the data, we have performed a principal component analysis (PCA), and the first 10 principal components (PCs) were calculated using PLINK [see Supplementary Figure S1 in our previous study (Ohi et al. 2020b)]. We have confirmed that there was no population stratification using PCs from the SNP array in our Japanese participants, and the PCs extracted from our participants were completely located on those extracted from the JPT (Japanese in Tokyo, Japan) population (Ohi et al. 2020b). Thus, we did not use the PCs to control for possible population stratification in this study. The marginal significance level for all statistical tests was set at *P*_*uncorr*_<0.05. To control for type I error due to multiple testing, we calculated the *P*_*GC*_ value corrected by genomic control (GC). GC is a method used to control for multiple comparisons in genetic association study testing multiple genetic variants (Devlin et al. 2001). The GC method utilizes the distribution of test statistics across all genetic variants to estimate the genomic inflation factor (*λ*), which reflects the extent of inflation in the test statistics due to population structure or other confounding factors. To implement GC, the test statistics from the individual SNP association tests are divided by the estimated *λ*. This adjustment effectively counteracts the inflation caused by population structure or other sources of systematic bias. By applying GC, more accurate *P* values that appropriately account for multiple comparisons can be obtained. The significance level in this study was set at *P*_*GC*_<0.05.

## Results

### Associations between mitochondrial genetic variants and schizophrenia, bipolar disorder, and psychotic disorders

In total, 42, 42, and 38 genetic variants in the mitochondrial chromosome remained after each QC in the PSY vs. HC, BD vs. HC, and SZ vs. HC cohorts, respectively. The mitochondrial allelic frequencies of all 45 genetic variants among patients with BD, patients with SZ, and HCs are summarized in Table 2. As shown in Table 2, all genetic variants that survived only in one of the comparisons (PSY vs. HC, BD vs. HC, or SZ vs. HC) were variants with lower MAF (e.g., 0.001–0.05) in patients or in HCs. LD relationships between mitochondrial genetic variants in each diagnostic group are provided in Supplementary Fig. 1. Overall, linkage disequilibrium (LD) patterns were similar among the three groups.

**Table 2:** Differences in mitochondrial allelic frequencies among patients with BD, patients with SZ, and HCs

			MAF (A1)				PSYs			BD			SZ		
Rs number	BP	A1/A2	PSYs	BD	SZ	HCs	OR	*P*_*uncorr*_ (*χ*^*2*^)	*P*_*GC*_	OR	*P*_*uncorr*_ (*χ*^*2*^)	*P*_*GC*_	OR	*P*_*uncorr*_ (*χ*^*2*^)	*P*_*GC*_
**rs28625645**	489	A/G	0.32	0.33	0.31	0.37	0.79	0.26 (1.3)	0.42	0.85	0.62 (0.2)	0.69	0.78	0.25 (1.3)	0.47
**rs3901846**	499	A/G	0.022	0.039	0.017	0.051	0.43	0.12 (2.4)	0.26	0.76	0.73 (0.1)	0.78	0.33	0.083 (3.0)	0.27
**rs3888511**	961	G/A	0.050	0.020	0.058	0.051	0.97	0.95 (< 0.1)	0.97	0.38	0.35 (0.9)	0.44	1.15	0.75 (0.1)	0.84
**rs111033179**	1005	G/A	-	0.039	-	0.005	-	-	-	8.00	**0.047 (4.0)**	0.10	-	-	-
**rs2000974**	1048	A/G	0.018	-	0.023	0.005	3.56	0.23 (1.5)	0.39	-	-	-	4.64	0.13 (2.3)	0.34
**rs111033358**	1382	C/A	0.081	0.059	0.087	0.10	0.78	0.46 (0.6)	0.59	0.55	0.35 (0.9)	0.44	0.85	0.64 (0.2)	0.77
**rs28358573**	1442	A/G	0.022	0.020	0.023	0.025	0.88	0.84 (< 0.1)	0.89	0.77	0.81 (0.1)	0.84	0.91	0.90 (< 0.1)	0.93
**rs200251800**	1709	A/G	0.009	0	0.012	0.025	0.35	0.19 (1.7)	0.35	0	0.25 (1.3)	0.35	0.45	0.33 (0.9)	0.54
**rs28358580**	2416	G/A	-	0.020	-	0.010	-	-	-	1.95	0.58 (0.3)	0.65	-	-	-
**rs200221487**	2772	A/G	0.090	0.020	0.11	0.066	1.39	0.37 (0.8)	0.52	0.28	0.20 (1.6)	0.29	1.76	0.13 (2.3)	0.33
**rs199713564**	2831	A/G	0.014	0.020	0.012	0.051	0.26	**0.029 (4.8)**	0.12	0.38	0.34 (0.9)	0.44	0.22	**0.035 (4.4)**	0.18
**rs3928306**	3010	A/G	0.39	0.37	0.40	0.32	1.36	0.14 (2.2)	0.29	1.23	0.52 (0.4)	0.60	1.39	0.13 (2.3)	0.33
**rs200999343**	3206	A/G	0.11	0.12	0.10	0.051	2.26	**0.033 (4.5)**	0.13	2.49	0.083 (3.0)	0.15	2.19	0.051 (3.8)	0.21
**rs2853516**	3316	A/G	0.014	0.020	0.012	0.010	1.34	0.75 (0.1)	0.82	1.95	0.58 (0.3)	0.65	1.15	0.89 (< 0.1)	0.93
**rs41460449**	3394	G/A	0.023	0.039	0.018	0.021	1.10	0.88 (< 0.1)	0.92	1.92	0.45 (0.6)	0.54	0.86	0.84 (< 0.1)	0.90
**rs201212638**	3398	G/A	0.018	-	-	0	-	0.058 (3.6)	0.17	-	-	-	-	-	-
**rs200319905**	3497	A/G	0.046	0.040	0.047	0.041	1.13	0.80 (0.1)	0.86	0.98	0.98 (< 0.1)	0.99	1.17	0.75 (0.1)	0.84
**rs3021088**	5460	A/G	0.045	0.059	0.041	0.071	0.62	0.25 (1.3)	0.41	0.82	0.76 (0.1)	0.80	0.56	0.21 (1.5)	0.43
**rs200165736**	6253	G/A	0.022	0.039	0.017	0.005	4.50	0.13 (2.2)	0.28	8.00	**0.047 (4.0)**	0.10	3.48	0.25 (1.3)	0.47
**rs28358884**	8414	A/G	0.39	0.37	0.40	0.32	1.36	0.13 (2.3)	0.28	1.26	0.48 (0.5)	0.56	1.39	0.13 (2.3)	0.33
**rs201336180**	8684	A/G	0.009	0.020	-	0.010	0.88	0.90 (< 0.1)	0.93	1.95	0.58 (0.3)	0.65	-	-	-
**rs2000975**	8701	A/G	0.34	0.37	0.33	0.40	0.76	0.18 (1.8)	0.34	0.89	0.71 (0.1)	0.76	0.73	0.14 (2.1)	0.35
**rs199646902**	9053	A/G	0.009	0	0.012	0.015	0.59	0.56 (0.3)	0.68	0	0.38 (0.8)	0.47	0.77	0.78 (0.1)	0.86
**rs201397417**	10,345	G/A	0.036	0.059	0.029	0.061	0.57	0.23 (1.4)	0.39	0.96	0.96 (< 0.1)	0.96	0.46	0.15 (2.1)	0.35
**rs2853826**	10,398	A/G	0.29	0.30	0.29	0.32	0.90	0.61 (0.3)	0.71	0.92	0.81 (0.1)	0.84	0.89	0.61 (0.3)	0.74
**rs200478835**	10,410	G/A	0.099	0.12	0.093	0.030	3.48	**5.17 × 10** ^**− 3**^ **(7.8)**	**0.045**	4.24	**9.70 × 10** ^**− 3**^ **(6.7)**	**0.034**	3.27	**0.011 (6.4)**	0.11
**rs200487531**	10,609	G/A	0.040	0.039	0.041	0.051	0.79	0.61 (0.3)	0.71	0.76	0.73 (0.1)	0.78	0.79	0.65 (0.2)	0.77
**rs200873900**	11,696	A/G	0.013	0	0.017	0.025	0.52	0.37 (0.8)	0.52	0	0.25 (1.3)	0.35	0.68	0.60 (0.3)	0.74
**rs28359169**	11,969	A/G	0.013	-	-	0.005	2.67	0.38 (0.8)	0.53	-	-	-	-	-	-
**rs2853501**	13,105	G/A	0.014	0.020	0.012	0.036	0.37	0.14 (2.2)	0.29	0.54	0.56 (0.3)	0.63	0.32	0.14 (2.2)	0.34
**rs200044200**	13,135	A/G	-	0.059	-	0	-	-	-	-	**6.15 × 10** ^**− 4**^ **(11.7)**	**4.9 × 10** ^**− 3**^	-	-	-
**rs28359178**	13,708	A/G	0.036	0.078	0.023	0.005	7.29	**0.030 (4.7)**	0.12	16.68	**8.94 × 10** ^**− 4**^ **(11.0)**	**6.4 × 10** ^**− 3**^	4.67	0.13 (2.3)	0.34
**rs200657506**	13,942	G/A	0.013	0.039	0.006	0.015	0.88	0.88 (< 0.1)	0.91	2.64	0.28 (1.2)	0.37	0.38	0.38 (0.8)	0.58
**rs28357671**	14,178	G/A	0.004	0	0.006	0.020	0.22	0.14 (2.2)	0.28	0	0.30 (1.1)	0.40	0.28	0.23 (1.4)	0.44
**rs201551481**	14,927	G/A	0.018	0.020	0.017	0.015	1.18	0.83 (< 0.1)	0.88	1.29	0.82 (< 0.1)	0.86	1.15	0.87 (< 0.1)	0.91
**rs200786872**	14,979	G/A	0.11	0.12	0.10	0.051	2.26	**0.033 (4.5)**	0.13	2.49	0.083 (3.0)	0.15	2.19	0.051 (3.8)	0.21
**rs2853506**	15,218	G/A	0.014	0	0.018	0.016	0.87	0.87 (< 0.1)	0.90	0	0.37 (0.8)	0.47	1.13	0.88 (< 0.1)	0.92
**rs201250154**	15,236	G/A	0.013	0.020	0.012	0.051	0.25	**0.027 (4.9)**	0.11	0.37	0.33 (0.9)	0.43	0.22	**0.034 (4.5)**	0.18
**rs527236176**	15,314	A/G	0.022	0.039	-	0.010	2.24	0.33 (1)	0.48	3.98	0.14 (2.2)	0.23	-	-	-
**rs527236177**	15,323	A/G	0.036	0.039	0.035	0.046	0.78	0.61 (0.3)	0.71	0.85	0.84 (< 0.1)	0.87	0.76	0.60 (0.3)	0.74
**rs199951903**	15,497	A/G	0.041	0.039	0.041	0.046	0.88	0.79 (0.1)	0.85	0.85	0.84 (< 0.1)	0.87	0.89	0.82 (0.1)	0.88
**rs527236193**	15,758	G/A	0.009	0	0.012	0.015	0.59	0.56 (0.3)	0.67	0	0.38 (0.8)	0.47	0.76	0.77 (0.1)	0.85
**rs201023973**	15,860	G/A	0.036	0.039	0.035	0.041	0.88	0.8 (0.1)	0.86	0.96	0.96 (< 0.1)	0.97	0.85	0.77 (0.1)	0.85
**rs193303003**	15,941	G/A	0.018	0.020	0.017	0.020	0.88	0.86 (< 0.1)	0.90	0.97	0.97 (< 0.1)	0.98	0.86	0.84 (< 0.1)	0.90
**rs41378955**	16,390	A/G	0.040	0.059	0.035	0.015	2.72	0.12 (2.4)	0.27	4.04	0.071 (3.3)	0.14	2.34	0.22 (1.5)	0.44

MAF, minor allele frequency; BP, biological position; OR, odds ratio; PSY, psychotic disorder; BD, bipolar disorder; SZ, schizophrenia

The reference genome sequence is GRCh38/hg38. *P*_*uncorr*_ values < 0.05 are shown in boldface. *P*_*GC*_ values < 0.05 are shown in boldface and underlined

**Fig. 1:**
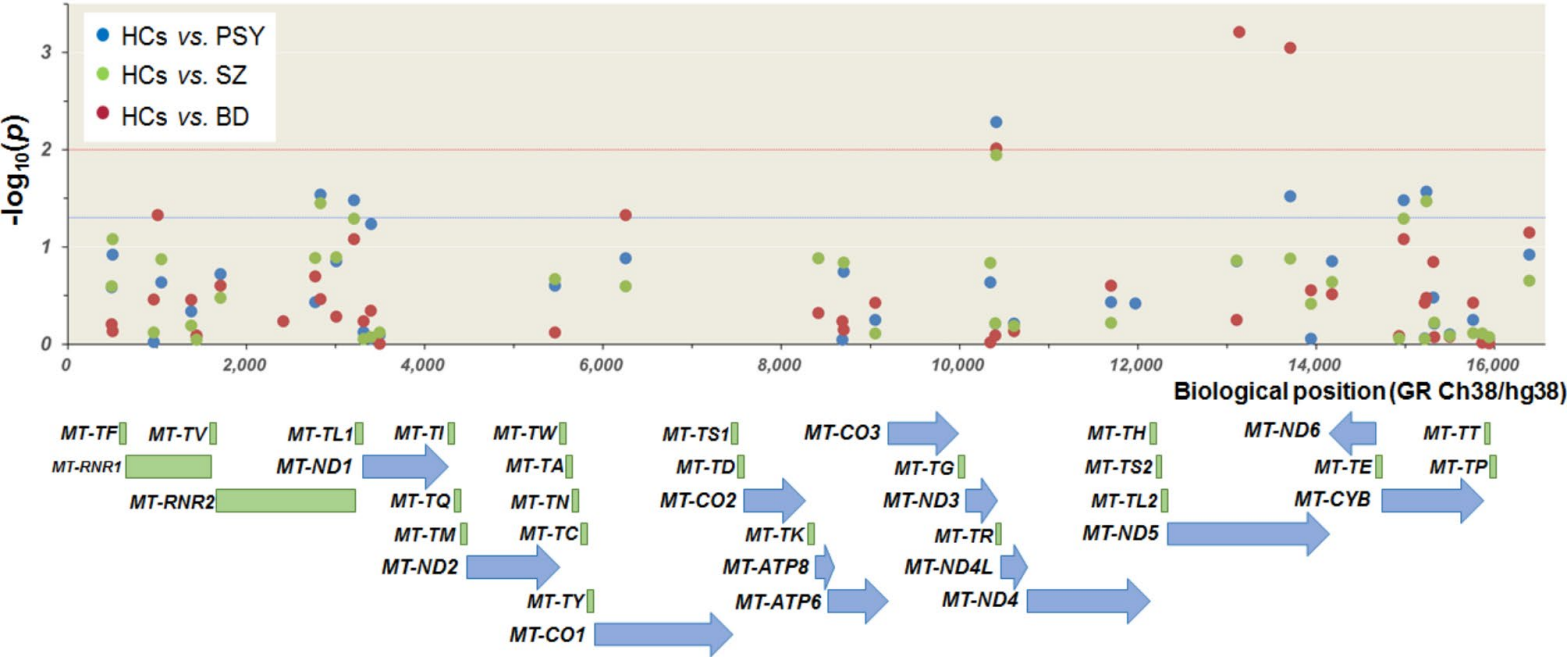
Associations of mitochondrial genetic variants with bipolar disorder, schizophrenia, and psychotic disorders. The red line indicates a *P* value of 0.01; the blue line indicates a *P* value of 0.05. Genes (blue arrow) and RNAs (green) in the mitochondrial chromosome are indicated. HCs, healthy controls; PSY, psychotic disorders; BD, bipolar disorder; SZ, schizophrenia

We first investigated genetic associations between mitochondrial genetic variants and PSY consisting of BD and SZ. Of 42 genetic variants, the allelic frequencies of six genetic variants (rs199713564, rs200999343, rs200478835, rs28359178, rs200786872, and rs201250154) differed between patients with PSY and HCs (Table 2; Fig. 1, *χ*^*2*^ = 4.5–7.8, *P*_*uncorr*_=0.033–5.17 × 10^− 3^). After correcting for multiple comparisons, only the association with rs200478835 was significant (*P*_*GC*_=0.045), with other associations being not significant (*P*_*GC*_>0.05). The MAF of the genetic variant (rs200478835) was higher in patients with PSY than in HCs.

We next investigated genetic associations of mitochondrial genetic variants with BD and SZ separately. Of 42 genetic variants, the allelic frequencies of five (rs111033179, rs200165736, rs200478835, rs200044200, and rs28359178) differed between patients with BD and HCs (Table 2; Fig. 1, *χ*^*2*^ = 4.0–11.7, *P*_*uncorr*_=0.047–6.15 × 10^− 4^). Even after correcting for multiple comparisons, three genetic variants (rs200478835, rs200044200 and rs28359178) were significantly associated with BD (*P*_*GC*_=0.034–4.9 × 10^− 3^), with no significant associations with the other two genetic variants (*P*_*GC*_>0.05). The MAFs of those genetic variants (rs200478835, rs200044200 and rs28359178) were higher in patients with BD than in HCs. Of the 38 genetic variants, the allelic frequencies of three (rs199713564, rs200478835, and rs201250154) differed between patients with SZ and HCs (Table 2; Fig. 1, *χ*^*2*^ = 4.4–6.4, *P*_*uncorr*_=0.035–0.011). However, there were no significant genetic variants associated with SZ after correcting for multiple comparisons (*P*_*GC*_>0.05).

### Case reports of BD patients who carry the minor G-allele of rs200044200

Individuals with the minor G-allele of *NADH dehydrogenase 5* (*ND5*) rs200044200 were found only among patients with BD (MAF = 0.059) and not in HCs (MAF = 0) [odds ratio (OR)=∞]. The detailed medical information of the three patients with BD is shown in Table 3.

**Table 3:** Case series of three BD patients with the minor G-allele of rs200044200

	BD patient 1	BD patient 2	BD patient 3
**Age (years)**	48	58	44
**Sex**	female	male	male
**Age at onset (years)**	25	44	35
**Duration of illness (years)**	23	14	9
**Family history**	SZ (aunt)	Unspecified psychiatric disorder (brother)	ASD/ADHD (son), Dementia (father)
**Types of BD**	I	II	II
**Developmental disorders**	-	-	-
**Years of education**	14	12	16
**Premorbid IQ**	92	110	104
**Present IQ**	80	n.a.	n.a.
**Present occupation**	Support for continuous employment type A	Unemployed	Unemployed
**Former occupation**	Children’s nurse	Bus driver	Clerical work
**Primary symptom**	Mania	Depression	Depression
**Psychotic episode**	+	-	-
**Rapid cycler**	+	-	+
**Present prescription (mg/day)**	Aripiprazole 12 mg, Zotepine 25 mg, Biperiden 2 mg, Clonazepam 2 mg, Loflazepic acid 2 mg, Flunitrazepam 4 mg, Triazolam 0.25 mg, Valproic acid 800 mg	Flunitrazepam 2 mg, Lithium 400 mg	Quetiapine 100 mg, Flunitrazepam 2 mg, Zopiclone 7.5 mg, Lithium 900 mg

BD, bipolar disorder; ASD, autism spectrum disorder; ADHD, attention-deficit hyperactivity disorder

The three patients commonly had neuropsychiatric family histories, though the neuropsychiatric diagnoses differed: patient 1, SZ (aunt); patient 2, unspecified psychiatric disorder (brother); and patient 3, autism spectrum disorder/attention-deficit hyperactivity disorder (son) and dementia (father). There were no other common characteristics, such as types of BD, developmental disorders or premorbid IQ, among these patients.

## Discussion

This is the first study to investigate associations of genome-wide genetic variants in the mitochondrial chromosome with BD, SZ and PSY in a Japanese population. Of mitochondrial genetic variants, five, three, and six were associated with BD, SZ and PSY, respectively. Of these variants, three (rs200478835, rs200044200 and rs28359178) and one (rs200478835) were significantly associated with BD and PSY, respectively, even after correcting for multiple comparisons. The minor alleles of rs200478835, rs200044200 and rs28359178 were commonly associated with risks of BD and PSY. Interestingly, the minor G-allele of rs200044200 was observed only in three patients with BD but not in HCs. The common feature of the three patients with BD was a neuropsychiatric family history. Our findings suggest that mitochondrial genetic variants may be associated with BD and PSY in both European and Japanese populations.

We found rs200478835, rs200044200 and rs28359178 to be associated with BD and rs200478835 with PSY in a Japanese population. In contrast, previous studies have not investigated associations of these genetic variants with BD or PSY in European populations (Gonçalves et al. 2018; Hagen et al. 2018; Hudson et al. 2014; Sequeira et al. 2012). MAFs of rs200478835, rs200044200 and rs28359178 in European populations are 0.0049, 0.0041, and 0.11, respectively (https://www.ncbi.nlm.nih.gov/snp/). Due to low MAFs, these studies might not have investigated associations in European populations. rs200478835 of the arginine-tRNA (*TRNR*) gene (protein noncoding) is located approximately 500 bp downstream of the *NADH dehydrogenase 3* (*ND3*) gene (protein coding) and approximately 2 kb upstream of the *NADH dehydrogenase 4* (*ND4*) gene (protein coding). rs200044200 and rs28359178 are both missense variants, A (Ala) > T (Thr) and A (Ala) > T (Thr), respectively, of the *ND5* gene.

NADH dehydrogenase is a membrane-associated protein localizing to mitochondrial membranes and is also known as complex I. NADH dehydrogenase is the enzyme that catalyzes the first reaction of the electron transfer system, which is vital for energy production. Previous studies have suggested that NADH dehydrogenase expression and activity in cells are decreased in patients with BD and SZ (Andreazza et al. 2010; Das et al. 2022; Holper et al. 2019). Furthermore, mutations in the *ND4* and *ND5* genes of the mitochondrial genome are associated with BD and SZ (Bamne et al. 2008; Frye et al. 2017; Torrell et al. 2013). These findings suggest that mitochondrial genetic variants in genes related to NADH dehydrogenase may contribute to the pathogenesis of BD and SZ via dysfunction of energy production.

As stated above, several previous studies have identified significant genetic associations between several mitochondrial genetic variants (rs28357375, rs28357968, rs527236209, rs869096886, rs1599988, rs2854131, rs2853503, rs2853504, rs193302985, rs2853506, rs3937033, rs2857291, rs28380140, rs3088053, and rs2853497) and BD, SZ or PSY in European populations (Gonçalves et al. 2018; Hagen et al. 2018; Hudson et al. 2014; Sequeira et al. 2012). Of these genetic variants associated with BD, SZ or PSY in European populations, the current study examined associations only for rs2853506 related to the risk of SZ in European populations with BD, SZ or PSY in a Japanese population after applying our QC. However, the minor G-allele of rs2853506 was not significantly associated with SZ in the Japanese population, even though the direction of the association was consistent between the present (OR = 1.13) and previous (OR ≈ 1.30) studies (Hagen et al. 2018). This finding suggests that some mitochondrial genetic variants might be commonly associated with risk of BD and SZ in different populations.

We found individuals with the minor G-allele of rs200044200 only among three patients with BD (MAF = 0.059) but not in HCs (MAF = 0) (OR=∞). Moreover, all the BD patients with the minor G-allele of rs200044200 (100%, 3/3) had several neuropsychiatric family histories, such as SZ, neurodevelopmental disorders, dementia, and unspecified psychiatric disorder. Of our all patients with BD, 33.3% (17/51) had neuropsychiatric family histories; 29.2% of patients with BD who did not have the minor G-allele of rs200044200 had neuropsychiatric family histories. The minor G-allele of rs200044200 was significantly associated with neuropsychiatric family histories in patients with BD (*P* = 0.033). Compared with that of rs200044200 in European populations (MAF = 0.0041), the MAF was higher in East Asian populations (MAF = 0.019). However, it has been reported that rs200044200 is benign for Leigh syndrome (https://www.ncbi.nlm.nih.gov/clinvar/RCV000854899/), which is a progressive neurodegenerative disorder caused by abnormalities in mitochondrial energy generation (Thorburn et al. 1993), even though the SNP is a missense variant. In contrast, it is unknown whether rs200044200 affects mitochondrial function in PSY, including BD. While the rs200044200 mitochondrial missense variant may be associated with an increased risk of BD in some populations, further research is needed to fully understand its role in the development of this condition and to determine how it may interact with other genetic and environmental factors.

### Limitations

There are some limitations to the interpretations of our findings. The sample size of our study was small compared to previous studies, potentially resulting in false-positive and -negative findings. Due to QC based on MAFs in our small sample size, we might have excluded some SNPs investigated in previous studies. Because our participants were recruited at a single institute, sample selection bias might have occurred. Further study with a larger sample size at multiple institutes in a Japanese population is needed. Although we investigated mitochondrial genetic variants in genomic DNA extracted from whole-blood samples, there might be not whole-blood-specific but brain-specific mitochondrial genetic variants in genomic DNA extracted from brain samples in patients with BD, SZ or PSYs.

## Conclusions

We investigated associations of BD, SZ and PSY with genome-wide genetic variants in the mitochondrial chromosome in a Japanese population. Of 45 genetic variants, three (rs200478835, rs200044200 and rs28359178) and one (rs200478835) were significantly associated with BD and PSY, respectively. Interestingly, the minor G-allele of rs200044200 was detected only in three patients with BD but not in HCs. The common feature of these patients with BD was neuropsychiatric family histories. Our findings suggest that mitochondrial genetic variants may be associated with BD and PSY in European populations as well as in the Japanese population.

## Electronic supplementary material

Below is the link to the electronic supplementary material.

Supplementary Material 1

## Data Availability

Our data are not publicly available because they contain information that could compromise the research participants’ privacy/consent.

## References

[CR1] Andreazza AC, Shao L, Wang JF, Young LT (2010). Mitochondrial complex I activity and oxidative damage to mitochondrial proteins in the prefrontal cortex of patients with bipolar disorder. Arch Gen Psychiatry.

[CR2] Bamne MN, Talkowski ME, Moraes CT, Manuck SB, Ferrell RE, Chowdari KV (2008). Systematic association studies of mitochondrial DNA variations in schizophrenia: focus on the ND5 gene. Schizophr Bull.

[CR3] Das SC, Hjelm BE, Rollins BL, Sequeira A, Morgan L, Omidsalar AA (2022). Mitochondria DNA copy number, mitochondria DNA total somatic deletions, Complex I activity, synapse number, and synaptic mitochondria number are altered in schizophrenia and bipolar disorder. Translational psychiatry.

[CR4] Devlin B, Roeder K, Wasserman L (2001). Genomic control, a new approach to genetic-based association studies. Theor Popul Biol.

[CR5] Frye MA, Ryu E, Nassan M, Jenkins GD, Andreazza AC, Evans JM (2017). Mitochondrial DNA sequence data reveals association of haplogroup U with psychosis in bipolar disorder. J Psychiatr Res.

[CR6] Giménez-Palomo A, Dodd S, Anmella G, Carvalho AF, Scaini G, Quevedo J (2021). The role of Mitochondria in Mood Disorders: from physiology to pathophysiology and to treatment. Front Psychiatry.

[CR7] Gonçalves VF, Giamberardino SN, Crowley JJ, Vawter MP, Saxena R, Bulik CM (2018). Examining the role of common and rare mitochondrial variants in schizophrenia. PLoS ONE.

[CR8] Grande I, Berk M, Birmaher B, Vieta E (2016). Bipolar disorder. Lancet (London England).

[CR9] Hagen CM, Gonçalves VF, Hedley PL, Bybjerg-Grauholm J, Bækvad-Hansen M, Hansen CS (2018). Schizophrenia-associated mt-DNA SNPs exhibit highly variable haplogroup affiliation and nuclear ancestry: bi-genomic dependence raises major concerns for link to disease. PLoS ONE.

[CR10] Holper L, Ben-Shachar D, Mann JJ (2019). Multivariate meta-analyses of mitochondrial complex I and IV in major depressive disorder, bipolar disorder, schizophrenia, Alzheimer disease, and Parkinson disease. Neuropsychopharmacology: official publication of the American College of Neuropsychopharmacology.

[CR11] Hudson G, Gomez-Duran A, Wilson IJ, Chinnery PF (2014). Recent mitochondrial DNA mutations increase the risk of developing common late-onset human diseases. PLoS Genet.

[CR12] Inada T, Inagaki A (2015). Psychotropic dose equivalence in Japan. J Neuropsychiatry Clin Neurosci.

[CR13] Kataoka Y, Shimada T, Koide Y, Okubo H, Uehara T, Shioiri T (2020). Differences in executive function among patients with schizophrenia, their unaffected first-degree relatives and healthy participants. Int J Neuropsychopharmacol.

[CR14] Kato T, Kunugi H, Nanko S, Kato N (2000). Association of bipolar disorder with the 5178 polymorphism in mitochondrial DNA. Am J Med Genet.

[CR15] Matsuoka K, Uno M, Kasai K, Koyama K, Kim Y (2006). Estimation of premorbid IQ in individuals with Alzheimer’s disease using japanese ideographic script (Kanji) compound words: japanese version of National Adult Reading Test. J Neuropsychiatry Clin Neurosci.

[CR16] McGuffin P, Rijsdijk F, Andrew M, Sham P, Katz R (2003). Cardno a the heritability of bipolar affective disorder and the genetic relationship to unipolar depression. Arch Gen Psychiatry.

[CR17] McMahon FJ, Stine OC, Meyers DA, Simpson SG, DePaulo (1995). JR patterns of maternal transmission in bipolar affective disorder. Am J Hum Genet.

[CR18] Morris G, Walder KR, Berk M, Marx W, Walker AJ, Maes M (2020). The interplay between oxidative stress and bioenergetic failure in neuropsychiatric illnesses: can we explain it and can we treat it?. Mol Biol Rep.

[CR19] Mosquera-Miguel A, Torrell H, Abasolo N, Arrojo M, Paz E, Ramos-Ríos R (2012). No evidence that major mtDNA european haplogroups confer risk to schizophrenia. Am J Med Genet Part B Neuropsychiatric genetics: official publication Int Soc Psychiatric Genet.

[CR20] Mullins N, Forstner AJ, O’Connell KS, Coombes B, Coleman JRI, Qiao Z (2021). Genome-wide association study of more than 40,000 bipolar disorder cases provides new insights into the underlying biology. Nat Genet.

[CR21] Munakata K, Tanaka M, Mori K, Washizuka S, Yoneda M, Tajima O (2004). Mitochondrial DNA 3644T–>C mutation associated with bipolar disorder. Genomics.

[CR22] Nishimura Y, Kanda Y, Sone H, Aoyama H (2021). Oxidative stress as a common key event in Developmental Neurotoxicity. Oxidative Med Cell Longev.

[CR30] Ohi K, Takai K, Kuramitsu A, Sugiyama S, Shioiri T, Common Brain. Cortical Abnormality in Smoking Behavior and Bipolar Disorder: Discriminant Analysis Using Cortical Thickness and Surface Area. Cerebral cortex (New York, N.Y.: 1991) 2022c. 10.1093/cercor/bhab490 35040937

[CR23] Ohi K, Shimada T, Nemoto K, Kataoka Y, Yasuyama T, Kimura K (2017). Cognitive clustering in schizophrenia patients, their first-degree relatives and healthy subjects is associated with anterior cingulate cortex volume. NeuroImage Clin.

[CR24] Ohi K, Shimada T, Kataoka Y, Koide Y, Yasuyama T, Uehara T (2019). Intelligence decline between present and premorbid IQ in schizophrenia: Schizophrenia non-affected relative project (SNARP). Eur neuropsychopharmacology: J Eur Coll Neuropsychopharmacol.

[CR25] Ohi K, Nishizawa D, Muto Y, Sugiyama S, Hasegawa J, Soda M (2020). Polygenic risk scores for late smoking initiation associated with the risk of schizophrenia. NPJ schizophrenia.

[CR26] Ohi K, Nishizawa D, Shimada T, Kataoka Y, Hasegawa J, Shioiri T (2020). Polygenetic risk scores for Major Psychiatric Disorders among Schizophrenia Patients, their first-degree relatives, and healthy participants. Int J Neuropsychopharmacol.

[CR27] Ohi K, Nishizawa D, Sugiyama S, Takai K, Kuramitsu A, Hasegawa J (2021). Polygenic risk scores differentiating Schizophrenia from Bipolar Disorder are Associated with Premorbid Intelligence in Schizophrenia Patients and healthy subjects. Int J Neuropsychopharmacol.

[CR28] Ohi K, Ishibashi M, Torii K, Hashimoto M, Yano Y, Shioiri T (2022). Differences in subcortical brain volumes among patients with schizophrenia and bipolar disorder and healthy controls. J Psychiatry Neurosci.

[CR29] Ohi K, Nishizawa D, Sugiyama S, Takai K, Fujikane D, Kuramitsu A et al. Cognitive performances across individuals at high genetic risk for schizophrenia, high genetic risk for bipolar disorder, and low genetic risks: a combined polygenic risk score approach. Psychol Med 2022b:1–10. 10.1017/S0033291722001271 35971752

[CR31] Ruderfer D, Ripke S, McQuillin A, Boocock J, Stahl E, Pavlides J (2018). Genomic dissection of bipolar disorder and Schizophrenia, including 28 subphenotypes. Cell.

[CR32] Ryu E, Nassan M, Jenkins GD, Armasu SM, Andreazza A, McElroy SL (2018). A genome-wide search for bipolar disorder risk loci modified by mitochondrial genome variation. Mol neuropsychiatry.

[CR33] Saha S, Chant D, Welham J, McGrath (2005). J a systematic review of the prevalence of schizophrenia. PLoS Med.

[CR34] Sequeira A, Martin MV, Rollins B, Moon EA, Bunney WE, Macciardi F (2012). Mitochondrial mutations and polymorphisms in psychiatric disorders. Front Genet.

[CR35] Smeland OB, Bahrami S, Frei O, Shadrin A, O’Connell K, Savage J (2020). Genome-wide analysis reveals extensive genetic overlap between schizophrenia, bipolar disorder, and intelligence. Mol Psychiatry.

[CR36] Steckert AV, Valvassori SS, Moretti M, Dal-Pizzol F, Quevedo J (2010). Role of oxidative stress in the pathophysiology of bipolar disorder. Neurochem Res.

[CR37] Sullivan PF, Kendler KS, Neale MC (2003). Schizophrenia as a complex trait: evidence from a meta-analysis of twin studies. Arch Gen Psychiatry.

[CR38] Thorburn DR, Rahman J, Rahman S. Mitochondrial DNA-Associated Leigh Syndrome and NARP. In: Adam MP, Everman DB, Mirzaa GM, Pagon RA, Wallace SE, Bean LJH, Gripp KW, Amemiya A, editors. GeneReviews(®). Seattle (WA): University of Washington, Seattle; 1993. Copyright. © 1993–2022, University of Washington, Seattle. GeneReviews is a registered trademark of the University of Washington, Seattle. All rights reserved.

[CR40] Torrell H, Montaña E, Abasolo N, Roig B, Gaviria AM, Vilella E (2013). Mitochondrial DNA (mtDNA) in brain samples from patients with major psychiatric disorders: gene expression profiles, mtDNA content and presence of the mtDNA common deletion. Am J Med Genet Part B Neuropsychiatric genetics: official publication Int Soc Psychiatric Genet.

[CR41] Trubetskoy V, Pardiñas AF, Qi T, Panagiotaropoulou G, Awasthi S, Bigdeli TB (2022). Mapping genomic loci implicates genes and synaptic biology in schizophrenia. Nature.

[CR42] Verge B, Alonso Y, Valero J, Miralles C, Vilella E, Martorell L (2011). Mitochondrial DNA (mtDNA) and schizophrenia. Eur psychiatry: J Association Eur Psychiatrists.

[CR43] Wolyniec PS, Pulver AE, McGrath JA, Tam D (1992). Schizophrenia: gender and familial risk. J Psychiatr Res.

[CR44] Wu Y, Chen M, Jiang J (2019). Mitochondrial dysfunction in neurodegenerative diseases and drug targets via apoptotic signaling. Mitochondrion.

[CR45] Xu FL, Ding M, Yao J, Shi ZS, Wu X, Zhang JJ (2017). Association between mitochondrial DNA variations and schizophrenia in the northern chinese Han population. PLoS ONE.

[CR46] Zhang W, Tang J, Zhang AM, Peng MS, Xie HB, Tan L (2014). A matrilineal genetic legacy from the last glacial maximum confers susceptibility to schizophrenia in Han Chinese. J Genet genomics = Yi chuan xue bao.

