# Supplementary material for: Mitochondrial genetic variants associated with bipolar disorder and Schizophrenia in a Japanese population
Source: Int J Bipolar Disord. 2023 Jul 21;11:26. doi: 10.1186/s40345-023-00307-6 (PMC10361950; doi:10.1186/s40345-023-00307-6)
Supplement: Supplementary file 1 — Supplementary Material 1 [file 40345_2023_307_MOESM1_ESM.docx]

**Supplementary information**

**Supplementary Fig. 1.** Pairwise linkage disequilibrium (*r^2^* and *D’*) among mitochondrial genetic variants in HCs, patients with SZ, and patients with BD.


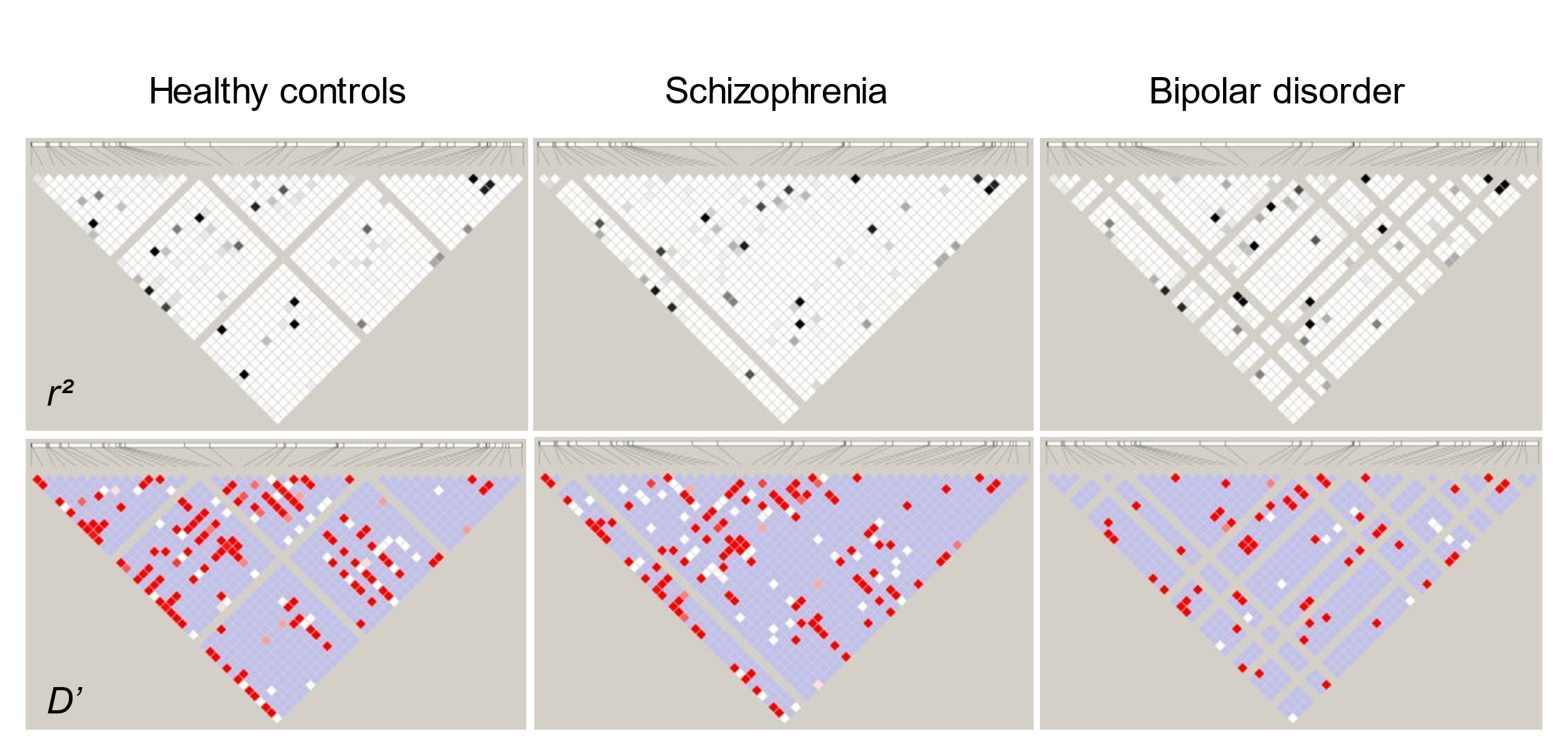


**Supplementary Table 1.** Identified mitochondrial genetic variants associated with BD, SZ and PSYs in previous studies, and comparisons of minor allele frequencies of these variants among European and East Asian populations.

|  | ***n*** | |  |  | **MAF** | |
| --- | --- | --- | --- | --- | --- | --- |
| **Study** | **Cases** | **Controls** | **Associated disorder** | **Identified SNP** | **European** | **East Asian** |
| **Sequeira *et al.* (2012)** | BD: 965 | 3,938 | BD | rs28357375 | 0.0090 | 0.018 |
|  |  |  | BD | rs28357968 | 0.0024 | 0.0015 |
|  | SZ: 1,137 | 3,938 | SZ | rs3937033 | 0.48 (global) | |
|  |  |  | SZ | rs2857291 | 0.042 (global) | |
|  | PSY: 2,102 | 3,938 | PSY | rs2857291 | 0.042 (global) | |
|  |  |  | PSY | rs28357968 | 0.0024 | 0.0015 |
|  |  |  | PSY | rs28380140 | 0.0018 | 0.037 |
|  |  |  | PSY | rs3088053 | 0.069 | 0 |
|  |  |  | PSY | rs2853497 | 0.0156 | 0 |
| **Gonçalves *et al.* (2018)** | SZ: 4,778 | l5,819 | SZ | rs527236209 | 0.20 | 0.0011 |
|  |  |  | SZ | rs869096886 | 0.17 | 0 |
|  |  |  | SZ | rs1599988 | 0.17 | 0 |
| **Hudson *et al.* (2014)** | SZ: 2,019 | 15,302 | SZ | rs2854131 | 0.088 | 0 |
|  |  |  | SZ | rs2853503 | 0.060 | 0 |
|  |  |  | SZ | rs2853504 | 0.037 | 0 |
|  |  |  | SZ | rs193302985 | 0.042 | 0.59 |
|  |  |  | SZ | rs2853506 | 0.039 | 0.030 |
| **Hagen *et al.* (2018)** | SZ: 2,538 | 23,743 | SZ | rs193302985 | 0.042 | 0.59 |
|  |  |  | SZ | rs2853506 | 0.039 | 0.030 |

BD, bipolar disorder; SZ, schizophrenia; PSY, psychotic disorder; MAF, minor allele frequency.
